# Antinociceptive and Anti-Inflammatory Effects of Zerumbone against Mono-Iodoacetate-Induced Arthritis

**DOI:** 10.3390/ijms17020249

**Published:** 2016-02-18

**Authors:** Ting-Yi Chien, Steven Kuan-Hua Huang, Chia-Jung Lee, Po-Wei Tsai, Ching-Chiung Wang

**Affiliations:** 1 Department of Food Science, Nutrition, and Nutraceutical Biotechnology, Shih Chien University, Taipei City 10462, Taiwan; swecon@g2.usc.edu.tw; 2 School of Pharmacy, College of Pharmacy, Taipei Medical University, Taipei City 11031, Taiwan; powei@tmu.edu.tw; 3 Division of Uro-Oncology, Department of Surgery, Chi Mei Medical Center, Tainan City 73657, Taiwan; skhsteven@yahoo.com.tw; 4 Department of Applied Life Science and Health, Chia Nan University of Pharmacy & Science, Tainan City 71710, Taiwan; 5 Ph.D. Program for Clinical Drug Discovery of Chinese Herbal Medicine, College of Pharmacy, Taipei Medical University, Taipei City 11031, Taiwan; cjlee@tmu.edu.tw; 6 Orthopedics Research Center, Taipei Medical University Hospital, Taipei City 11031, Taiwan

**Keywords:** *Zingiber zerumbet* Smith, zerumbone, arthritis, anti-inflammatory, heme oxygenase-1, metalloproteinase

## Abstract

The fresh rhizome of *Zingiber zerumbet* Smith (Zingiberaceae) is used as a food flavoring and also serves as a folk medicine as an antipyretic and for analgesics in Taiwan. Zerumbone, a monocyclic sesquiterpene was isolated from the rhizome of *Z. zerumbet* and is the major active compound. In this study, the anti-inflammatory and antinociceptive effects of zerumbone on arthritis were explored using *in vitro* and *in vivo* models. Results showed that zerumbone inhibited inducible nitric oxide (NO) synthase (iNOS), cyclooxygenase (COX)-2 expressions, and NO and prostaglandin E_2_ (PGE_2_) production, but induced heme oxygenase (HO)-1 expression in a dose-dependent manner in lipopolysaccharide (LPS)-stimulated RAW 264.7 cells. When zerumbone was co-treated with an HO-1 inhibitor (tin protoporphyrin (SnPP)), the NO inhibitory effects of zerumbone were recovered. The above results suggest that zerumbone inhibited iNOS and COX-2 through induction of the HO-1 pathway. Moreover, matrix metalloproteinase (MMP)-13 and COX-2 expressions of interleukin (IL)-1β-stimulated primary rat chondrocytes were inhibited by zerumbone. In an *in vivo* assay, an acetic acid-induced writhing response in mice was significantly reduced by treatment with zerumbone. Furthermore, zerumbone reduced paw edema and the pain response in a mono-iodoacetate (MIA)-induced rat osteoarthritis model. Therefore, we suggest that zerumbone possesses anti-inflammatory and antinociceptive effects which indicate zerumbone could be a potential candidate for osteoarthritis treatment.

## 1. Introduction

*Zingiber zerumbet* Smith, a wild ginger that belongs to the ginger family (Zingiberaceae), is used as a remedy to alleviate stomachaches, fevers, sores, and inflammation in Southeast Asian countries [1]. Zerumbone is a monocyclic sesquiterpene compound isolated from rhizomes of *Z. zerumbet*. Recently, some scientific research on the bioactivities of zerumbone, which was identified as a major compound of *Z. zerumbet*, reported that zerumbone possesses many pharmacological activities, such as chemoprevention [2,3], anti-inflammatory [4] and antiallergic activities [5].

Osteoarthritis (OA) also known as degenerative arthritis, is a chronic inflammatory disease characterized by cartilage loss, synovial swelling, and osteophyte formation. Presently, there is no ideal medical for the prevention or treatment of OA [6]. OA occur frequently in middle-aged and older people. Chief complaints of this disease are cardinal signs of inflammation which lead to knee joint deformities and ultimately crippling. When joint cartilage wear and tear, it usually accompanied with pain. Once the condition worsens, bones can rub against each other, causing even more pain and loss of movement.

Nitric oxide (NO) is one of the inflammatory mediators that plays important roles in inflammatory processes and destruction of articular cartilage. The functions of NO in inflammatory processes are to increase vasodilation and vascular permeability and stimulate white blood cells to release interleukin (IL)-1 and tumor necrosis factor (TNF). The roles of NO in the destruction of articular cartilage are to induce apoptosis, inhibit chondrocyte proliferation, inhibit synthesis of extracellular components including collagen and proteoglycan, inhibit IL-1 receptor antagonist synthesis, and stimulate matrix metalloproteinases (MMPs) which lead to increased articular cartilage destruction. According to previous study [7], MMP-1 and MMP-13 play rate-limiting roles in mediating the joint collagen degradation during OA progression. MMP-1 is mainly secreted by synovial cells and MMP-13 expression by chondrocytes in the cartilage. Therefore, we used MMP-13 as the biomarker of IL-1 induced chondrocytes degradation. 

Several experimental models mimicking the lesions of OA have been developed to evaluate potential anti-arthritic agents. The mono-iodoacetate (MIA) is frequently used as alkylating reagents to modify thiol groups in protein by *S*-carboxymethylation, and have been used to inhibit glycolysis. When MIA local injects into joints, it will immediately induce inflammation, disrupts chondrocyte metabolism and then produces cartilage degeneration. Moreover, MIA-induced OA rats is a minimally invasive animal model with cartilage and bone pathological features similar to those of human OA [8]. Also, the model of intra-articular iodoacetate injection provides a pre-clinical tool and an analysis of pain related behaviors in OA rats [9]. Collagenase activity significantly increased in the knee cartilage of rats injected with iodoacetate further results in progressive loss of chondrocytes [10]. The progression and severity of the articular lesions in this model can be easily controlled by the dose of MIA. A high dose of MIA injection into the knees of rats can rapidly produce severe OA symptoms which enables the *in vivo* study of pathophysiology and chondroprotective effects. Therefore, we used MIA as an inducer of OA animal model, analyzed HO-1 initiated iNOS and COX2/PGE_2_ signaling pathways to explore the inflammatory mechanism of zerumbone, and used MMP-13 as a biomarker of chondroprotective effects.

Many studies were performed to elucidate the biological activities of zerumbone and demonstrated its many pharmacological activities. However, there are few reports or investigations of its effects on OA. Thus, we conducted *in vivo* and *in vitro* experiments using macrophages, rat chondrocytes, and an MIA-induced OA model.

## 2. Results

### 2.1. Anti-Inflammatory Effects of Zerumbone on LPS-Induced RAW 264.7 Cells

**Figure 1:**
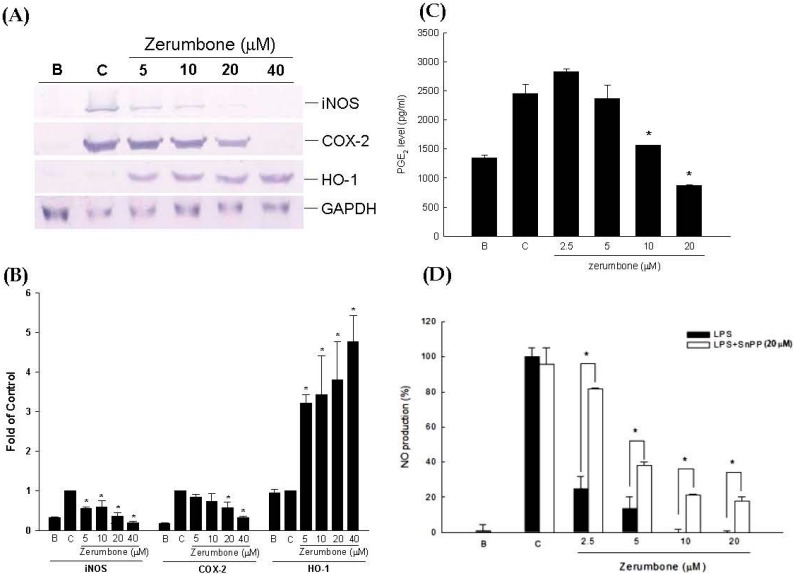
Anti-inflammatory action of zerumbone on lipopolysaccharide (LPS)-stimulated RAW 264.7 cells after treatment for 6 h. (**A**) Inducible nitric oxide (NO) synthase (iNOS), cyclooxygenase (COX)-2, and heme oxygenase (HO)-1 protein expressions; (**B**) Quantitational and statistical analysis of protein expressions, * *p* < 0.05 compared to control; (**C**) prostaglandin E_2_ (PGE_2_) production level, * *p* < 0.05, the zerumbone group compared to the control group; (**D**) NO production inhibition of zerumbone co-treated with tin protoporphyrin (SnPP), * *p* < 0.05, the zerumbone group compared to the group co-treated with SnPP. C: control, cells were pretreated with vehicle and LPS (500 ng/mL) B: blank, the cells incubated with vehicle alone. Data are summarized and expressed as Mean ± SEM of three individual experiments (*n* = 3).

Since iNOS and COX are mediators of inflammatory reactions, expressions of iNOS and COX-2 proteins in RAW 264.7 cells were assessed by a Western blot assay. Results showed that zerumbone suppressed iNOS and COX-2 protein expressions by lipopolysaccharide (LPS)-induced RAW 264.7 cells in a dose-dependent manner (Figure 1A,B). Subsequently, NO and PGE_2_ levels were measured from harvested medium. LPS-induced RAW 264.7 cells were treated with various concentrations of zerumbone (2.5~20 μM), and NO and PGE_2_ production showed significant decreases (Figure 1C,D). On the contrary, the anti-inflammatory modulator, heme oxygenase (HO)-1, was significantly upregulated by zerumbone in a dose-dependent manner. Treatment with zerumbone suppressed inflammatory reactions. As shown in Figure 1D, after exposure of cells to zerumbone with tin protoporphyrin (SnPP; 20 μM), a selective inhibitor of HO-1, the inhibitory effects of NO production were reversed. Cell viabilities were not affected in the presence of 20 μM zerumbone as determined by an MTT assay (data not shown).

### 2.2. Inhibition of COX-2 and MMP-13 Expressions by Zerumbone in IL-1β-stimulated PRCs

**Figure 2:**
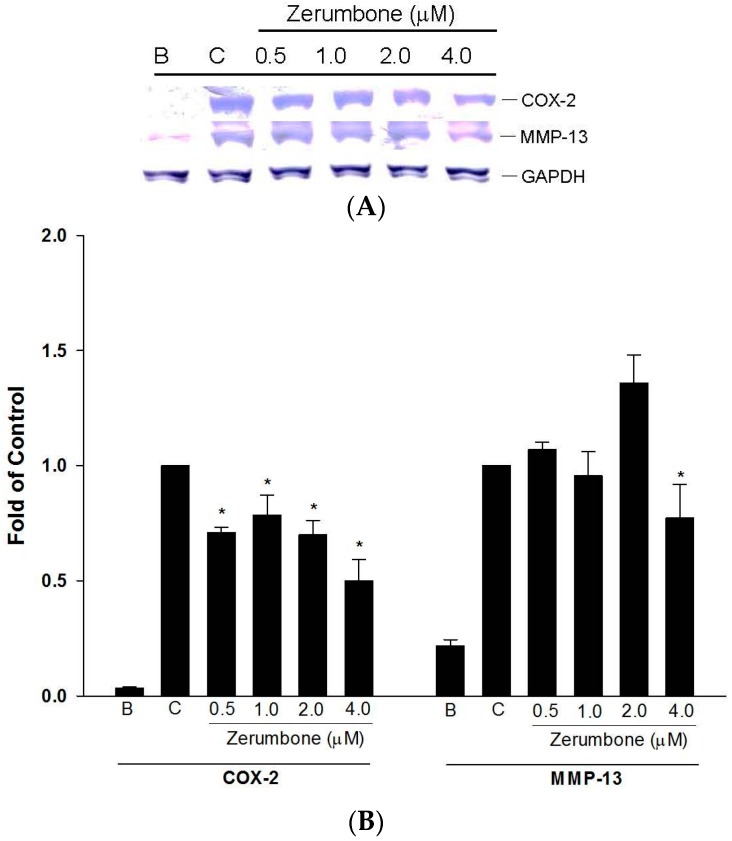
(**A**) Effects of zerumbone inhibiting matrix metalloproteinase (MMP)-13 expressions on interleukin (IL)-β-induced chondrocytes; (**B**) Quantitational and statistical analysis of protein expressions, * *p* < 0.05 compared to control; Data were used from three separate experiments; a picture of one is shown. C: control, cells were pretreated with vehicle and IL-1β (10 ng/mL) B: blank.

In arthritis, COX-2 promotes the production of prostaglandins which are important mediators of inflammatory pain and regulate catabolic processes in the cartilage. Also, MMPs are important factors in chondrolytic processes that contribute to degenerative changes in OA cartilage. The inhibitory effects of zerumbone on COX-2 and MMP-13 were evaluated using IL-1β-stimulated primary rat chondrocytes (PRCs). PRCs were treated with IL-1β (10 ng/mL) in the presence or absence of zerumbone at various concentrations (0.5~4 μM). Zerumbone significantly downregulated COX-2 and MMP-13 expressions by IL-1β-induced PRCs (Figure 2A,B).

### 2.3. Analgesic Effects of Zerumbone on the Acetic-Acid-Induced Writhing Response

**Figure 3:**
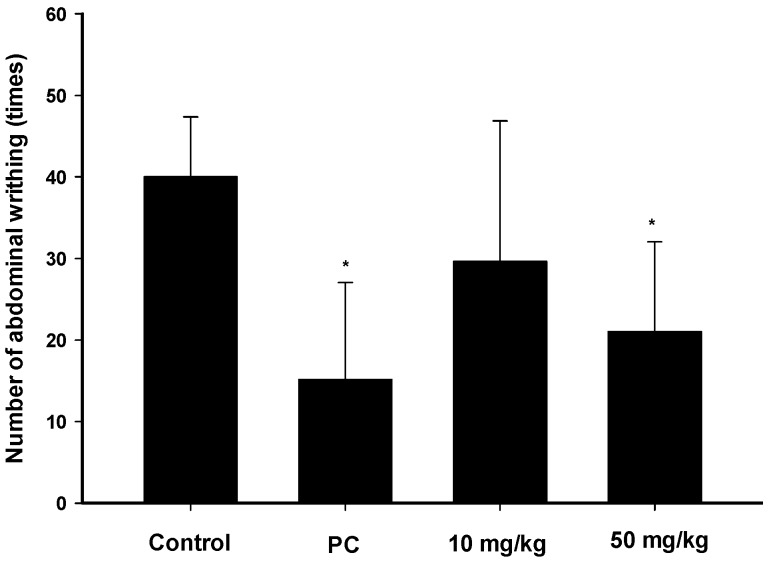
Abdominal writhing response of acetic-acid induces in mice. There were six mice per group. Results are expressed as Mean ± SEM. The positive control (PC) was morphine (5 mg/kg). * *p* < 0.05, compared to the control group.

The acetic writhing test is widely used for analgesic screening for potential peripheral analgesic effects of compounds. Figure 3 shows the cumulative amount of abdominal stretching responses of acetic acid-induced pain. A higher dosage (50 mg/kg) of zerumbone treatment significantly inhibited the number of writhing instances compared to the controls (*p * <  0.05). The inhibition by zerumbone was similar to that produced by morphine (Figure 3). This result indicates that the analgesic effect of zerumbone might be mediated by its peripheral effect.

### 2.4. Analgesic and Anti-Inflammatory Effects of Zerumbone on MIA-Induced OA

**Figure 4:**
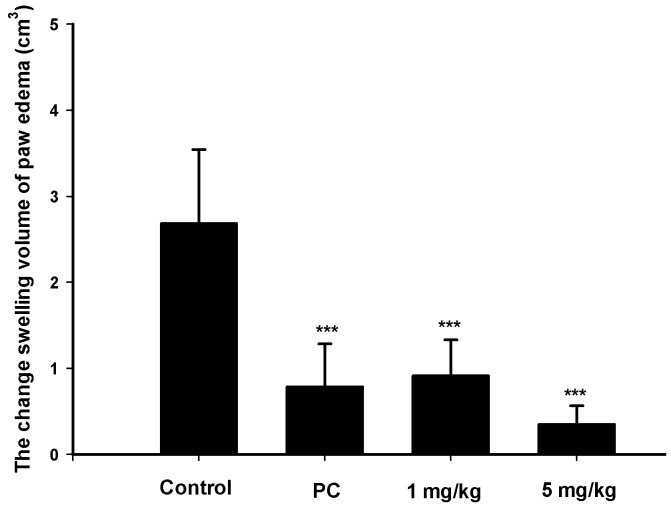
Change in the swelling volume due to paw edema after a mono-iodoacetate (MIA) injection in the ankle of a rat on days 1 to 4. The positive control (PC) was indomethacin (10 mg/kg). *** *p* < 0.0005 compared to the control.

Since zerumbone demonstrated anti-inflammatory effects *in vitro*, an MIA-induced OA model was used to further evaluate its effect on paw-edema *in vivo*. Edema was induced by an MIA injection in normal saline into the right-hind ankle of each rat on day 1. Paw volumes were first measured before the MIA injection on day 1 before the MIA injection as the baseline and then measured again on day 4. The increase in paw volume was calculated by the difference in paw volume on days 1 and 4. The swelling volume of the paw significantly increased in the control group. Both zerumbone and indomethacin markedly reduced paw edema compared to the control group. Further, a high dose (5 mg/kg) of zerumbone exhibited a greater suppressive effect than low-dose treatment (Figure 4).

**Figure 5:**
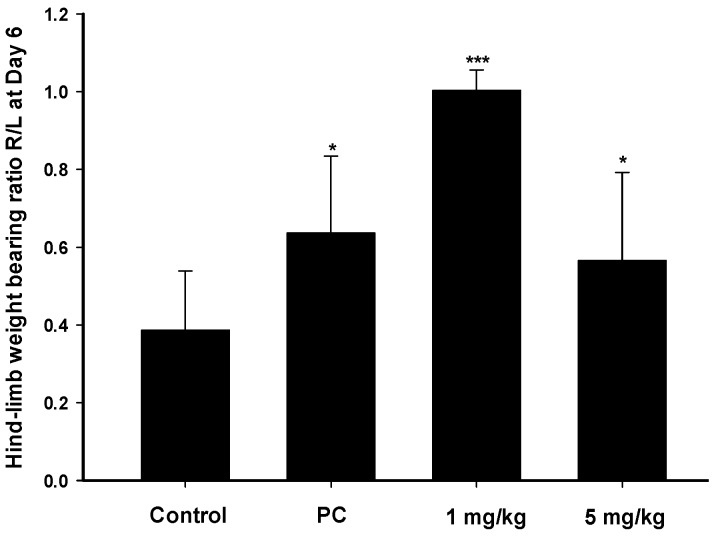
Distribution ratio of right and left hind-limb weight-bearing with mono-iodoacetate (MIA)-induced rat osteoarthritis on day 6. The positive control (PC) was indomethacin (10 mg/kg). * *p* < 0.05, *** *p* < 0.0005 compared to the control group.

Measurement of weight bearing in MIA-induced animal is an indicator of disease progression OA and reveal the efficacy of anti-inflammatory compounds [11]. Changes in the hind-limb weight distribution between the right (MIA-induced side) and left limbs were utilized as an index of joint discomfort in the osteoarthritic ankle. Weight distributions of rats on day 6 were assessed with an incapacitance tester which determined the distribution ratio of hind-limb weighting. Zerumbone at 1 and 5 mg/kg was orally administered daily, and Figure 5 indicates that a statistically significant difference existed between the imbalanced rate in control subjects and the imbalanced rates with zerumbone at both the high and low doses. The pharmacological activity of zerumbone were observed that reduced joint discomfort in this model which implied the therapies ability to intervention by a commonly utilized therapeutic agent.

## 3. Discussion

Chronic joint pain, such as with OA, is one the most common types of pain and is predicted to become the fourth leading cause of disability worldwide by 2020 [12]. OA is a loss of cartilage that initiates chondrocyte activation, and collagenases, such as MMPs and inflammatory factors, are released from the matrix. This regenerative process results in local activation of an inflammatory response. Without an ideal cure, medication management toward pain and symptom relief in clinical such as acetaminophen, non-steroidal anti-inflammatory drugs (NSAIDs), and glucosamine sulfate [13]. It was believed that the anti-inflammatory and analgesic effects of NSAIDs are due to the inhibition of prostaglandin synthesis [14]. However, NSAIDs are associated with a spectrum of side effects such as gastric ulceration, renal insufficiency, and prolonged bleeding times [15]. Thus, finding new and efficient pharmacological agents from medicinal plants for treating OA might be a solution. Several studies reported that zerumbone possesses remarkable antimicrobial, antihyperglycemic, antiallergic, anti-inflammatory, and chemopreventive activities [1]. Essential oil from the rhizome of *Z. zerumbet* exhibited a significant antinociceptic effects on acetic acid-induced writing test in a dose-dependent manner [16]. Perimal *et al.* demonstrated that zerumbone possesses significant peripheral and central antinociceptive effects [17,18]. However, previous research used an intraperitoneal injection to deliver zerumbone to experimental animals. Our present results revealed that oral administration of zerumbone also produced similar antinociceptive activities. In the present study, anti-inflammatory effects of zerumbone were assessed, and its suppression of COX-2 expression by LPS-induced RAW 264.7 cells was via a mechanism that might involve the upregulation of HO-1. HO-1 is known as an inducible isozyme with major anti-inflammatory effects mediated by the catalytic breakdown of proinflammatory free heme and production of the anti-inflammatory compounds, carbon monoxide and bile pigments [19]. Also, LPS-induced COX-2 and PGE_2_ expressions were inhibited by HO-1 through interruption of the toll-like receptor 4 (TLR4)/MyD88/nuclear factor (NF)-κB pathway [20]. In addition, OA cartilage expresses elevated levels of COX-2, with consequent increases in PGE_2_ production which contribute to synovial inflammation. These data suggest that the mechanism of zerumbone against OA may be an attenuation of COX-2/prostaglandin production by chronically inflamed tissues.

Numerous studies showed that the l-arginine/NO/cyclic guanosine monophosphate cascade participates in nociceptive processes [21]. Substances capable of inhibiting NO donors increase the analgesic effects of opioid receptor agonists during peripheral inflammation [22]. Articular chondrocytes stimulated with cytokines and/or endotoxin *in vitro* release various inflammatory mediators such as NO and PGE_2_ [23]. NO releasing was proposed that cause cartilage damage from OA-affected patient cartilage *ex vivo* [24]. Another research study demonstrated that NO induced chondrocyte death signaling including PGE_2_ production via COX-2 induction. Our results demonstrated that zerumbone was a potent NOS inhibitor, and significantly reduced NO production by LPS-stimulated macrophages. It not only involves the anti-inflammatory effects but possibly also the antinociceptive activity.

On the other hand, proinflammatory cytokines (such as IL-1β) damage cartilage via the synthesis and secretion of MMPs, which lead to matrix degradation. Rousset *et al.* identified a mechanism by which the induced expression of HO-1 downregulates ROS production by NADPH oxidase in human chondrocytes, that consequently reduces MMP-1 secretion and cell death, two main features of OA [25]. We found that zerumbone suppressed IL-1β-induced MMP-13 expression, which suggests that it might be influenced by downregulation of HO-1 as well.

Animal models of joint pain are mostly induced by an intra-articular injection of irritants or cartilage-degenerating agents. We use acetic acid-induced writhing mice and MIA-induced OA rats to evaluate the analgesic properties of zerumbone. Pain induced by acetic acid is caused by increased prostaglandins level which can be quantitatively determined in peritoneal fluid. For this reason, excessive prostaglandin production through COX are highly associated to the pain response [26]. This work showed that the efficacy to reduce the abdominal writhing of high dose zerumbone was comparable to morphine. Furthermore, the analgesic effect of zerumbone might be correlated to down-regulation of COX/prostaglandins signaling pathway.

The most obvious clinical symptoms of OA is joint swelling which is formed with inflammation of a synovial tissue and synovial fluid accumulation [27]. In the present study, we emphasized that the strong anti-inflammatory activity of zerumbone was mediated by HO-1. Decreases in PGE_2_ and NO production were also observed when macrophages were treated with zerumbone during the LPS challenge. Taken together, our results suggest that zerumbone not only produced a dose-dependent inhibition of the mice pain response in MIA-induced weight bearing imbalance, but was also able to reduce edema formation of mice.

In conclusion, this is the first known report demonstrating that zerumbone from *Z. zerumbet* has a markedly analgesic effect on MIA-induced OA in rats. We provided *in vitro* and *in vivo* evidence regarding the anti-inflammatory effectiveness relative to OA by zerumbone which exhibited inhibitory effects on NO and PGE_2_ production via HO-1, iNOS, and COX-2 modulation. Also, MMP production by IL-1β-induced PRCs was downregulated by the expression of COX-2, suggesting the potential chondroprotective activity of zerumbone. These pharmacological properties suggested that zerumbone is an ideal nutritional supplement for arthritis-associated inflammation.

## 4. Materials and Methods

### 4.1. Zerumbone

**Figure 6:**
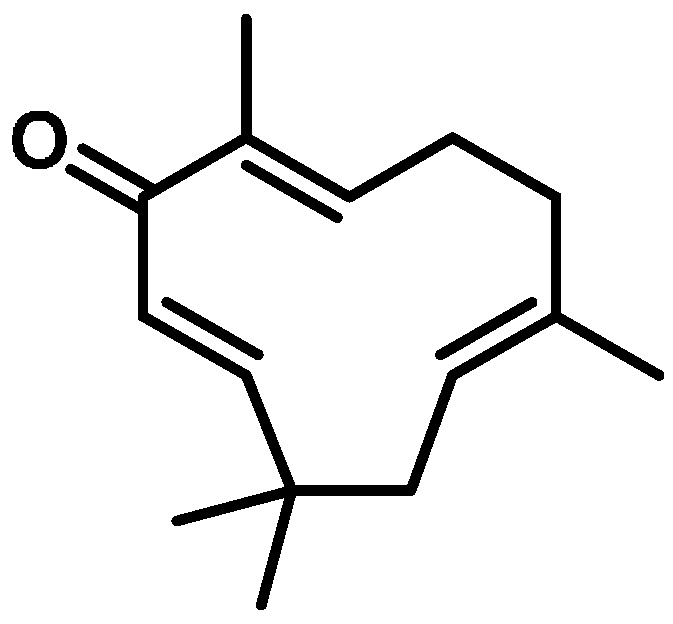
Chemical structure of zerumbone isolated from the fresh rhizome of *Zingiber zerumbet*.

Zerumbone ((2*E*,6*E*,10*E*)-2,6,9,9-tetramethylcycloundeca-2,6,10-trien-1-one), with an *M*w of 218 (Figure 6), was isolated and purified from rhizomes of *Z. zerumbet*, as in our previous study [28]. The purity of zerumbone was determined by high-performance liquid chromatography (HPLC), and was shown to exceed 99.0%.

### 4.2. Anti-Inflammatory in Vitro Assay

#### 4.2.1. Cell Cultures

The murine macrophage cell line, RAW 264.7 was purchased from American Type Culture Collection (Rockville, MD, USA). Cells were cultivated in Dulbecco’s modified Eagle’s medium (DMEM) supplemented with 10% fetal bovine serum (FBS), 100 IU/mL penicillin and 100 μg/mL streptomycin (Gibco BRL, Grand Island, NY, USA) in a humidified incubator containing 5% CO_2_ at 37 °C.

#### 4.2.2. Primary Chondrocyte Culture

Primary rat chondrocytes (PRCs) were obtained after the cartilage tissue of a rat knee joint was sequentially digested with pronase (10 g/L, Roche, Indianapolis, IN, USA) for 30 min and collagenase type IV (1 g/L, Sigma, St. Louis, MO, USA) for 6 h, as described previously [29]. Experiments were performed with 3^rd^ passage cells. Monolayer cultures were established in 60-mm Petri dishes at a concentration of 6 × 10^6^ cells/mL in DMEM supplemented with 10% FBS, 100 μg/mL streptomycin, and 100 IU/mL penicillin (Gibco BRL, Grand Island, NY, USA). PRCs were incubated in a humidified atmosphere of 95% air and 5% CO_2_ at 37 °C. Experiments were performed with 3rd passage cells.

#### 4.2.3. Measurement of NO and PGE_2_ Production

The measurement of NO and PGE_2_ was assessed as previously described [30]. Briefly, after 24 h of incubation with or without samples and/or LPS (500 ng/mL), cells generated NO and PGE_2_ in the culture medium. Quantitation of NO production was detected spectrophotometrically at 530 nm after the Griess reaction. The NO inhibition % was calculated using the following equation: NO inhibition (%) = [1 − (T/C)] × 100%; where T and C represent the mean optical density of LPS-stimulated RAW 264.7 cells with and without samples, respectively. Otherwise, the culture medium was collected after 24 h of incubation with a sample, and PGE_2_ concentrations were determined with an enzyme-linked immunosorbent assay (ELISA) kit (Amersham Pharmacia Biotech, Buckinghamshire, UK).

#### 4.2.4. Measurement of iNOS, COX-2, and MMP-13 Protein Expressions

IL-1β (10 ng/mL) in phosphate-buffered saline (PBS) was used to induce MMP-13 and COX-2 expressions in PRCs. Whole-cell lysates from cells treated with a sample were prepared by washing with PBS and lysing with radioimmunoprecipitation assay (RIPA) buffer. Equal amounts of protein (30 μg) from cell lysates were boiled for 5 min in sodium dodecylsulfate polyacrylamide gel electrophoresis (SDS-PAGE) sample buffer, separated by 10% SDS-PAGE, transferred to nitrocellulose membranes, and visualized using a BCIP/NBT kit (Gibco BRL, Grand Island, NY, USA). Protein expressions were analyzed with the Azure Biosystem C300 Imaging System. GAPDH expression was used as the internal control to compare with iNOS, COX-2 and HO-1, and the fold was calculated by the control group expression.

### 4.3. Anti-Inflammatory and Antinociception in Vivo Assay

#### 4.3.1. Animals

Male SD rats weighing 180~220 g and ICR mice weighing 20~25 g were housed in a controlled environment at 21 ± 2 °C with sufficient food and water and kept on an alternating 12-h dark and light cycle. The animal experiments were approved by Ethical Regulations on Animal Research of Taipei Medical University (approval no: LAC-100-0043).

#### 4.3.2. Acetic Acid-Induced Writhing Test

Acetic acid-induced writhing in ICR mice was induced according to a previously described method [31]. ICR mice were divided into four groups of six animals per group (*n* = 6). Rats in the control group were administered 10% DMSO (10 mL/kg *i.p.*) while animals in the zerumbone groups were administered 10 or 50 mg/kg zerumbone. Thirty minutes after treatment, each mouse was administered 10 mL/kg of 0.6% acetic acid (*i.p.*) before being assessed for 30 min inside its cage (25 × 25 × 30 cm^3^). The number of times writhing was displayed by each mouse was counted and recorded. Morphine (1 mg/kg, *i.p.*) served as the reference drug in the positive control group.

#### 4.3.3. MIA-Induced OA Model

**Figure 7:**
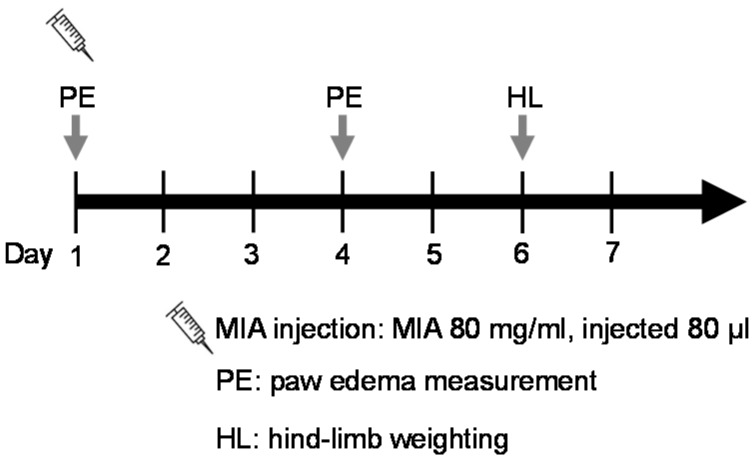
The experimental schedule of MIA-induced osteoarthritis model.

Four groups of six male SD rats each were divided as follows: control group, positive control group (PC), and zerumbone groups. OA in male SD rats was induced by an intra-articular injection of 80 μL of 80 mg/mL MIA (Sigma) into a rat’s ankle using a 100-μL syringe (Figure 7). Rats were randomly divided into six animals per group. After the MIA injection, the zerumbone groups were orally administered 1 or 5 mg/kg daily for 7 days. This is an effective dosage determined by our previous report [4]. The control group was treated with 10 mg/kg indomethacin daily for 7 days. The change in the paw volume was measured with a plethysmometer (Ugo Basile, Comerio VA, Italy) on days 1 and 4 after the MIA injection. Weight-bearing of both hind limbs was observed by an incapacitance tester with a dual-channel weight averager (Linton Instrumentation, Norfolk, UK) [11]. The weight-bearing force measured by the hind limb was averaged over a 3-s period. Each data point was the mean of three duplicate readings. The distribution ratio of weight-bearing of the right (MIA injection side) and left hind paws (control side) was assessed by the following equation: mean weight of the right hind paw/mean weight of the left hind paw.

### 4.4. Statistics

Results are shown as the Mean ± SEM. The data were analyzed using SPSS 17.0 software (SPSS, Inc., Chicago, IL, USA). Group differences were statistically assessed by one-way analysis variance (ANOVA), followed by the Fisher’s least significant different (LSD) test for comparison of the means. A *p*-value of < 0.05 was considered statistically significant.
